# Mixed gangliocytoma-pituitary neuroendocrine tumour: clinical, immunohistochemical, and molecular genetic profiles in a series of four patients

**DOI:** 10.1186/s40478-026-02225-x

**Published:** 2026-01-30

**Authors:** Konstantinos Dalakas, Britt Edén Engström, Abdellah Tebani, Thomas Olsson Bontell, Alice Larsson, Helena Nord, Cecilia Lindskog, Fredrik Pontén, Henning Bünsow Boldt, Oskar Ragnarsson, Olivera Casar-Borota

**Affiliations:** 1 Department of Endocrinology, Sahlgrenska University Hospital, Gothenburg, Sweden; 2 Department of Internal Medicine and Clinical Nutrition, Institute of Medicine, Sahlgrenska Academy, University of Gothenburg, Gothenburg, Sweden; 3 Department of Medical Sciences, Endocrinology and Mineral Metabolism, Uppsala University, Uppsala, Sweden; 4 Science for Life Laboratory, Department of Protein Science, KTH-Royal Institute of Technology, Stockholm, Sweden; 5 Department of Metabolic Biochemistry, UNIROUEN, AIMS, SysMedLab, CHU Rouen, Referral Center for Lysosomal Diseases, Filière G2M, Normandie University, Rouen, France; 6 Department of Clinical Pathology, Sahlgrenska University Hospital, Gothenburg, Sweden; 7 Department of Physiology, Institute of Neuroscience and Physiology, Sahlgrenska Academy, University of Gothenburg, Gothenburg, Sweden; 8 Department of Immunology, Genetics and Pathology, Uppsala University, Uppsala, Sweden; 9 Princess Máxima Center for Pediatric Oncology, Utrecht, The Netherlands; 10 Oncode Institute, Utrecht, The Netherlands; 11 Clinical Genomics Uppsala, Science for Life Laboratory, Uppsala University, Uppsala, Sweden; 12 Department of Pathology, Odense University Hospital, Odense, Denmark; 13 Department of Clinical Research, University of Southern Denmark, Odense, Denmark; 14 Wallenberg Center for Molecular and Translational Medicine, University of Gothenburg, Gothenburg, Sweden; 15 Department of Clinical Pathology, Uppsala University Hospital, Uppsala, Sweden

**Keywords:** Mixed gangliocytoma-pituitary adenoma, Mixed gangliocytoma-PitNET, PANCH, Acromegaly, Cushing’s disease

## Abstract

The vast majority of tumours in the sellar region are pituitary neuroendocrine tumours, also called pituitary adenomas. Sellar gangliocytomas (GCs), benign tumours that originate from neuronal ganglionic cells, account for less than 1% of sellar tumours. Even rarer are mixed gangliocytoma-pituitary neuroendocrine tumours (GC-PitNET). These tumours are often associated with hormone hypersecretion, most commonly resulting in acromegaly. The histogenesis of mixed GC–PitNET is currently unclear. In this paper, we present comprehensive clinical, immunohistochemical, targeting enrichment next generation DNA sequencing, and genome-wide methylation data from four patients with mixed GC-PitNETs, three with acromegaly and one with Cushing’s disease. Transcriptomic data are also included for two of the patients. Our findings indicate that mixed GC-PitNETs have different clinical course, with the acromegaly patients showing greater resistance to pharmacological therapy, as well as different protein expression and molecular features compared to respective pure PitNETs. The transcriptomic data on two patients with somatotroph GC-PitNET show involvement of mitochondrial and ribosomal genes, suggesting a distinct gene expression pattern, in comparison with pure somatotroph tumours. Furthermore, the expression pattern of selected stem cell markers, mainly SOX9, supports a common origin of the neuroendocrine and ganglionic tumour components, suggesting the involvement of stem cells in tumorigenesis.

**Supplementary Information:**

The online version contains supplementary material available at 10.1186/s40478-026-02225-x.

## Introduction

Tumours of the sellar region account for approximately 15% of all intracranial tumours. The vast majority of these are pituitary neuroendocrine tumours or pituitary adenomas, originating from adenohypophysial cells [1]. Sellar gangliocytomas, benign tumours that differentiate towards neuronal ganglionic cells, account for less than 1% of sellar tumours. Even more uncommon are tumours composed of both neuronal and neuroendocrine components [2, 3]. These have previously been designated as gangliocytoma-pituitary adenomas, though other terms such as pituitary adenoma-neuronal choristoma (PANCH) have also been used [4]. Throughout this manuscript, we use the term mixed gangliocytoma-pituitary neuroendocrine tumour (GC-PitNET) in accordance with the current WHO classification of pituitary tumours [3].

Mixed GC-PitNETs show a female predominance, and most cases are associated with growth hormone (GH) hypersecretion, typically caused by sparsely granulated somatotroph tumours, followed by hyperprolactinaemia [2, 3, 5–7]. Only a few patients with Cushing’s disease caused by a mixed GC-PitNET have been reported [2, 5–9]. In a systematic review, immunohistochemical analysis of the neuroendocrine component of 106 mixed tumours revealed positivity for GH and prolactin (PRL) in 43% of the cases, isolated GH in 33%, isolated PRL in 14%, adrenocorticotropic hormone (ACTH) in 6%, and ACTH in combination with PRL in 1% [2].

The predictive role of somatostatin receptor (SSTR) expression in somatotroph tumours is well known [10–13]. However, there are no reports on the correlation between immunohistochemical expression of somatostatin receptors and the response to somatostatin analogue (SSA) therapy in patients with acromegaly caused by mixed GC-PitNETs.

The histogenesis of mixed GC–PitNET is currently unclear, and three main theories have been proposed. According to an early theory, hypothalamic releasing-hormones produced by the intrasellar gangliocytic component promote the neoplastic growth of pituitary neuroendocrine cells [14]. The second theory suggests that ganglionic tumour cells develop through neuronal differentiation of the neuroendocrine cells [15]. Finally, a common origin of neuroendocrine and neuronal tumour cells has been proposed based on the expression of neurofilament protein [16], the neuronal marker NeuN [17], and pituitary specific positive transcription factor 1 (PIT1), a transcription factor regulating the development of somatotroph, lactotroph and thyrotroph cells, in both tumour components [18].

Recent studies have shown the expression of the stem cell markers sex-determining region Y-box 2 and 9 (SOX2 and SOX9) among both hormone-secreting PitNETs, including GH-secreting ones [19, 20] and clinically silent gonadotroph and corticotroph tumours [21]. However, the expression of stem cell markers and the potential role of stem cells in the development of mixed GC-PitNETs have not been explored.

Global mRNA profiling [22–24] and DNA methylation studies have been conducted on different types of PitNETs [23–26]. However, transcriptome and methylome data, as well as targeting enrichment DNA sequencing data, have not been reported in mixed GC-PitNETs so far. Nevertheless, molecular data on mixed GC-PitNETs would be valuable to determine the origin and development of these uncommon and complex neoplasms, a question that is still largely unanswered.

In this paper, we describe the clinical, histopathological, immunohistochemical, and molecular data from four patients diagnosed with mixed GC-PitNETs, three with acromegaly and one with Cushing’s disease. We discuss the clinical course and the response to medical therapy, with a specific focus on the response to SSAs and its correlation with the expression of somatostatin receptors. The presence of stem cell markers SOX2 and SOX9 suggests a potential role of stem cells in the pathogenesis of mixed GC-PitNETs. Transcriptomic analysis of tumours from two patients, and targeting enrichment sequencing and genome-wide DNA methylation profiling in all four patients provide deeper insights into the molecular features of the tumours, revealing distinct gene expression patterns that differ from the more common “non-mixed” PitNETs.

## Materials and methods

### Patient cohort

Four patients were included in the study, three with acromegaly and one with Cushing’s disease. All patients underwent transsphenoidal surgery for a sellar tumour with MRI features consistent with PitNETs. Clinical data, as well as laboratory data before and after surgery, and pharmacological therapy are presented in Table 1. Surgical specimens, consisting of formalin-fixed paraffin embedded (FFPE) blocks containing representative tumour tissues, were available for all patients. The diagnosis of mixed GC-PitNETs was confirmed through microscopic examination of standard haematoxylin-eosin (HE) stained sections and an immunohistochemical panel used as a routine diagnostic workup for PitNETs.

**Table 1:** Patient cohort: summary of the clinical, biochemical, and radiological characteristics, treatment and outcome

	Tumour size (mm) and parasellar invasiveness*	Pre-op IGF-1 (xULN)	Presurgical treatment	Postsurgical treatment	Follow-up
Patient 1—Cushing’s disease	28 × 30 × 30 Grade 4 left Grade 0 right	–	No	Proton beam therapy Ketoconazole (ongoing)	On Ketoconazole 48 months after radiotherapy
Patient 2—Acromegaly	17 × 18 × 21 Grade 0 bilaterally	3.1	No	Lanreotide Pituitary reoperation (after 7 yrs) because of increasing IGF-1 despite treatment with Lanreotide	On Lanreotide and Pegvisomant 12 months after reoperation with mildly elevated IGF-1
Patient 3—Acromegaly	35 × 30 × 35 Grade 4 left Grade 0 right	4.1	Lanreotide, ineffective. Pegvisomant, effective.	Pasireotide, ineffective Proton beam therapy Pegvisomant, effective	Biochemic control on Pegvisomant 57 months after radiotherapy
Patient 4—Acromegaly	30 × 25 × 35 Grade 1 left Grade 4 right	2.8	No	Lanreotide, ineffective Proton beam therapy Pegvisomant, effective	Biochemical control on Pegvisomant 62 months after radiotherapy

*According to Knosp

### Immunohistochemical (IHC) analyses

Immunohistochemical analyses for tumour classification and assessment of cell proliferation were performed according to the routine protocols, using the DAKO EnVision FLEX system and DAKO Autostainer. Normal pituitary gland served as a positive control for immunohistochemical analyses with antibodies targeting anterior pituitary hormones and pituitary-specific transcription factors (Steroidogenic factor 1 (SF1), PIT1 and T-box transcription factor (TPIT)). The Ki67 proliferation index was assessed by calculating the percentage of Ki67-immunolabelled cells per 2000 tumour cells in representative tumour areas. No Ki67 hot spots foci were identified in any of the tumours.

Immunohistochemical analyses of SSTRs were performed using monoclonal antibodies as previously described [13]. Endocrine pancreatic tissue was used as a positive control for IHC analysis with antibodies targeting SSTR types 1, 2, 3 and 5. SSTR expression was quantified using the immunoreactive score (IRS; range, 0–12), i.e. the product of the proportion of immunoreactive cells (0 = 0%, 1 ≤ 10%, 2 = 10–50%, 3 = 51–80%, and 4 ≥ 80%) and staining intensity (0 = no staining, 1 = weak, 2 = moderate, and 3 = strong). Both membranous and cytoplasmic staining were considered, though a considerable cytoplasmic staining was observed only for SSTR3, whereas other types of SSTRs showed distinct membranous immunolabelling.

For analyses of stem cell markers, immunohistochemical staining was performed on the Lab Vision Autostainer 480 S Module (Thermo Fisher Scientific, Fremont, CA) using antibodies targeting SOX2, SOX9, and paired-like homeodomain transcription factor 1 (PROP1). Normal pituitary gland tissue served as a positive control. Non-specific background staining was blocked using Ultra V, and the staining was visualised using an HRP-labelled peroxidase polymer and DAB as a chromogen.

A list of all antibodies used in the study is included in Supplemental Table 1.

### Transcriptomic analysis

Fresh frozen tumour tissue was available from patients 3 and 4 through the U-CAN project (www.u-can.uu.se). The full protocol for transcriptomic analysis has been described previously [22]. Briefly, three 10 μm thick sections from each frozen tissue block were collected, and total RNA was extracted using the RNeasy Mini Kit (Qiagen, Hilden, Germany). RNA sequencing was performed using the llumina TruSeq Stranded mRNA kit. The libraries were sequenced on the Illumina NovaSeq 6000 instrument. To obtain quantification scores for all human genes and transcripts across samples, transcript expression levels were calculated as transcript per million (TPM) by mapping processed reads to the human reference genome GRCh37/hg19 ref and with gene models based on Ensembl (v92) using Kallisto (v.0.43.1). Thereafter, gene expression levels were calculated by summing up all the TPM values of all alternatively spliced protein-coding transcripts of the corresponding gene, amounting to a total of 19,670 protein-coding genes. All TPM values were TMM normalised between all the samples. An expression level cut-off was set at 1 TPM. Data analysis and visualisation were performed using R (version 4.0.0). The full TPM data matrix is presented in Supplemental Table 2. Gene Set Enrichment Analysis (GSEA) for pathway analysis was performed in Enrichr software based on top 20 genes, ranked on within-sample TPM (Supplemental Table 3).

### Target enrichment next-generation sequencing

Genomic DNA was extracted from FFPE tissue blocks with representative tumour tissue containing > 80% of the tumour cells using QIAamp DNA FFPE tissue kit (Qiagen, Hilden, Germany). KAPA Biosystems HyperPlus reagents (ROCHE, Basel, Switzerland) in combination with Twist GMS560 gene panel probes and target enrichment reagents (Twist Bioscience, San Francisco, CA, USA) were used for preparation of sequencing ready-made libraries. The libraries were paired-end sequenced (150 cycles) on a NovaSeqX Plus system (Illumina San Diego, CA, USA).

The GMS560 gene panel covers approximately 1.6 megabase (Mb) coding sequence of 544 genes (Supplemental Table 4) that allows the possibility of detecting Single Nucleotide Variants (SNVs) and Insertions and Deletions (INDELS). The GMS560 panel is also suitable for both tumour mutational burden (TMB)- and microsatellite instability (MSI)-score calculations. Mutations can be detected at variant allele frequency (VAF) ≥ 2% in validated clinical hotspot positions, and at VAF ≥ 5% across the rest of the panel content in samples with a total read coverage of ≥ 50 M and a median target coverage of ≥ 500x. The design, optimization and validation are the result of a nationwide collaborative effort within the Genomic Medicine Sweden (GMS) Solid Tumor work package (https://genomicmedicine.se/wpcontent/uploads/2023/11/GMS560_synopsis.pdf).

Bioinformatic analyses were performed using the GMS Twist Solid Pipeline constructed using Hydra Genetics modules. Hard filtered VCF files for each sample were uploaded to QIAGEN Clinical Insight Interpret (QCI) for interpretation of the variants. Additional filtering of variants was applied in QCI.

BAM files for each sample were uploaded to Integrated Genomics Viewer (IGV) to facilitate visualization of each variant from the QCI output.

### Genome-wide DNA methylation profiling

DNA was isolated from FFPE tissues using the GeneRead DNA FFPE Kit (Qiagen, Germany), following the manufacturer’s instructions. DNA methylation profiling was conducted using the Infinium MethylationEPIC Kit (Illumina, USA), also referred to as 850k analysis, as the methylation status of 850,000 CpG sites are interrogated with this array. The quantity and quality of the sample DNA met the requirements recommended by Illumina for FFPE material. The workflow included DNA restoration with the Infinium HD FFPE DNA Restore Kit. Complete 850k datasets were collected for patients 3 and 4, while patients 1 and 2 had array detection rates of 90% and 80%, respectively. Methylome data analysis, including classification and copy number variation (CNV) calculation, was performed using the online classifier tool (version 12.8) at https://app.epignostix.com/ [27] with a decision threshold of ≥ 0.9 considered match and thus “classified”.

## Results

### Clinical cases

The age of the four patients ranged from 40 to 69 years, 1 male and 3 females.

Patient 1 presented with fatigue and subjective visual impairment. Initially, there were no hormonal abnormalities or visual field defects. Due to progression of the tumour, the patient underwent surgery three years after the initial presentation. During an annual follow-up, the patient had developed mild Cushing’s syndrome, confirmed biochemically, and radiotherapy was administered. Patients 2–4, presented with acromegalic features and increased Insulin-like Growth Factor 1 (IGF-1) and GH. One patient received pharmacological therapy before surgery. None of the patients were cured by surgery, and all required additional pharmacological therapy, radiotherapy and/or a second surgery (Table 1).

### Histopathological and immunohistochemical assessment

**Fig. 1:**
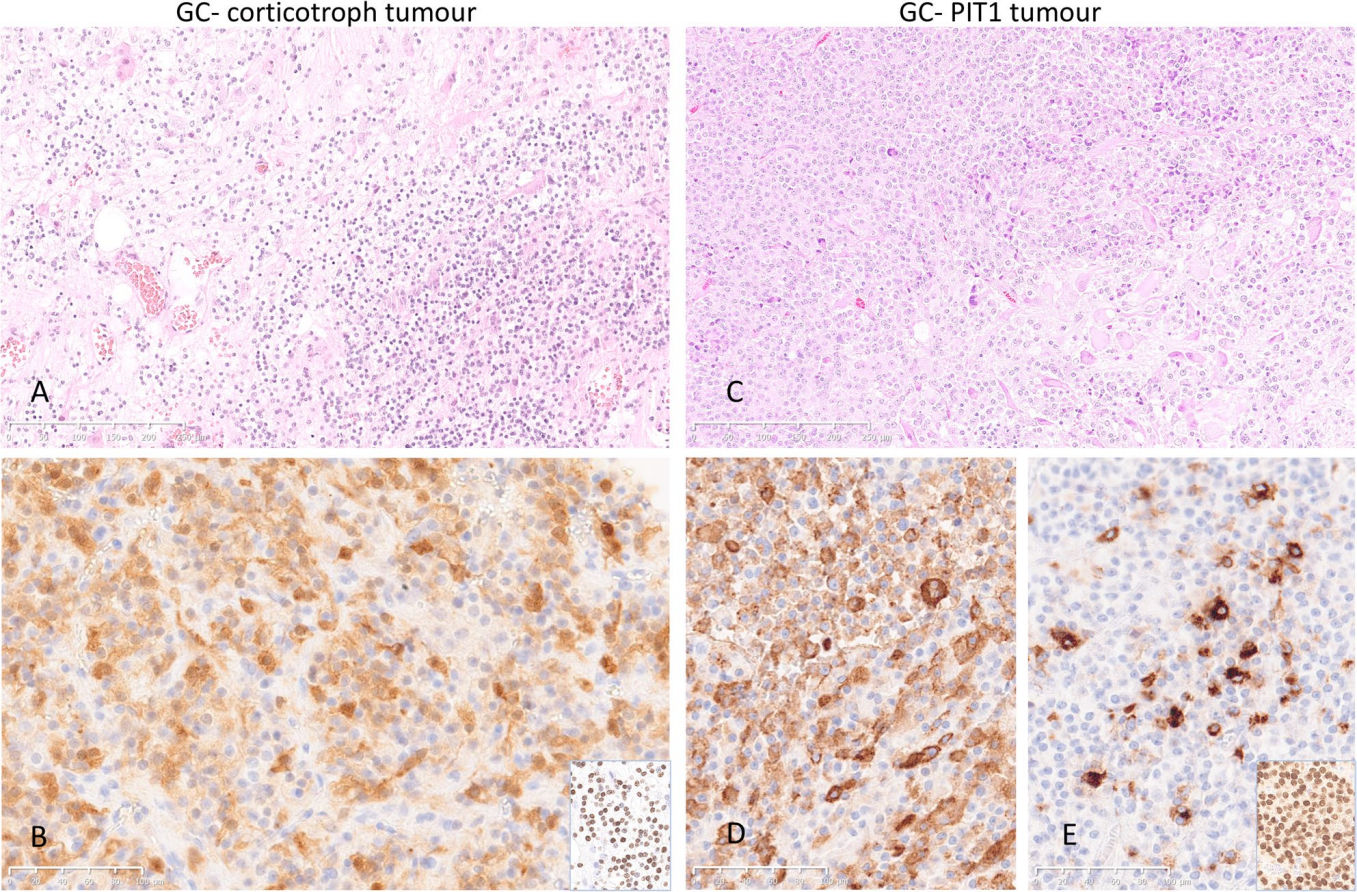
Histopathological appearance and immunohistochemical expression of the pituitary cell lineage markers in the two representative tumours, mixed GC-corticotroph tumour from patient 1 and mixed GC-PIT1 tumour from patient 3. Neuroendocrine and ganglionic cells are intermingled in the GC-corticotroph tumour specimen, as shown in the HE-stained slide (**A**). ACTH immunolabeling (**B**) and TPIT expression (insert in B) confirm the corticotroph origin of the neuroendocrine tumour cells. In the mixed GC-PIT1 tumour, ganglionic tumour cells are clustered as demonstrated in the HE-stained slide (**C**). Immunolabelling of GH (**D**), Prolactin (**E**) and PIT1 (insert in E) confirms that the neuroendocrine cells belong to the PIT1 cell lineage. (Magnification 100x for the HE slides and 200x for IHC labelled slides)

**Fig. 2:**
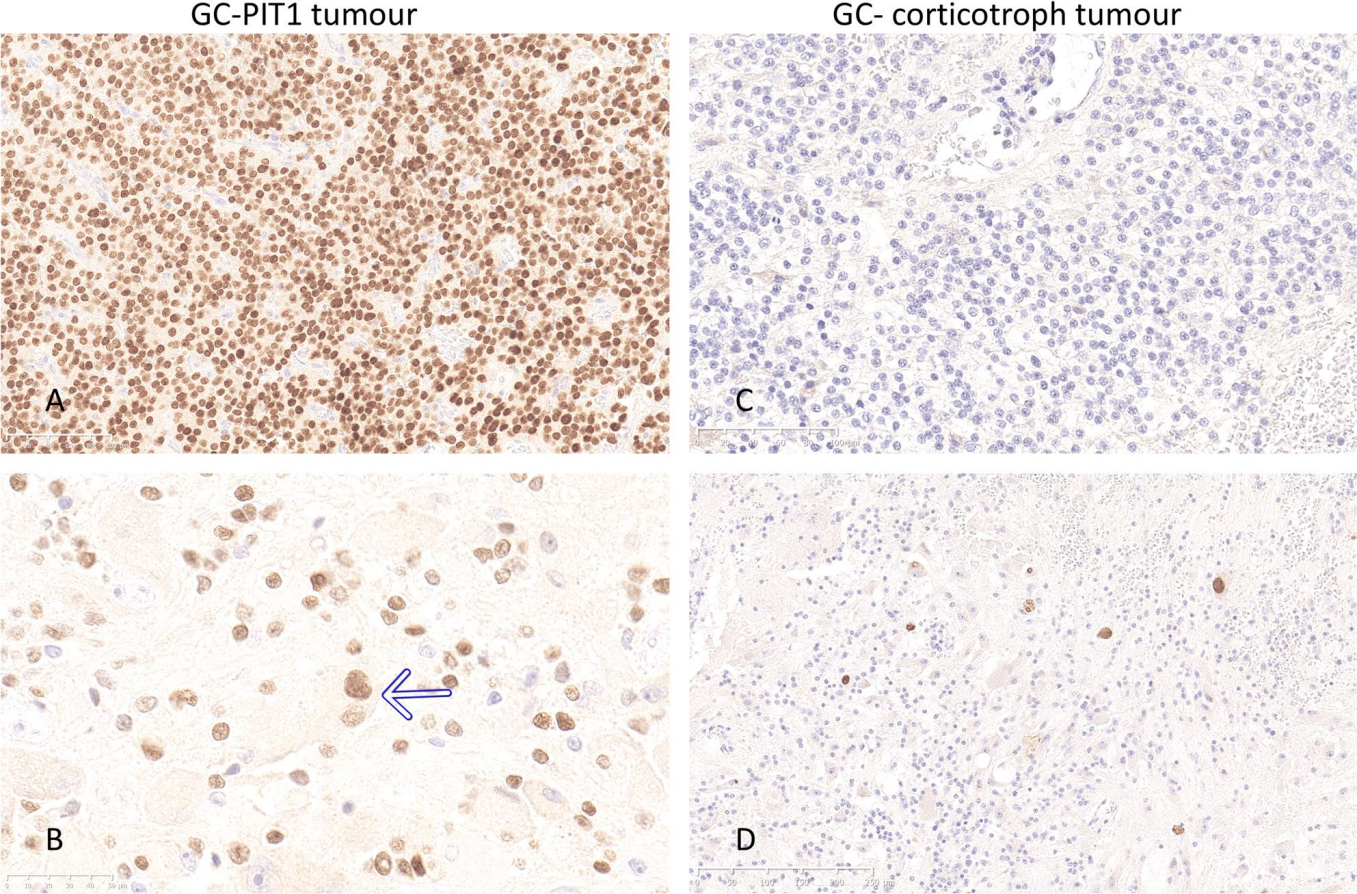
Expression of the transcription factor PIT1 in the mixed GC-PIT1 tumour from patient 3 (left) and GC-corticotroph tumour from patient 1 (right). PIT1 was strongly positive in all somatotroph cells (**A**) and in a subset of the ganglionic cells (**B**) in the mixed GC-PIT1 tumour; the arrow points to a binuclear ganglionic cell with nuclear PIT1 positivity. In the mixed GC-corticotroph tumour, there was no PIT1 immunolabelling in the neuroendocrine tumour cells (**C**); however, a proportion of ganglionic cells was distinctly positive (**D**). (Magnification 200x in A and C and 400x in B and D)

In all cases, histopathological features corresponded to mixed GC-PitNET. In patient 1, ganglionic cells were intermingled with ACTH and TPIT-positive corticotroph tumour cells. In the other patients, ganglionic cells formed clusters within somatotroph tumours showing variable expression of GH and prolactin in the PIT1 positive tumour cells in patients 2 and 3, whereas Patient 4 had a pure somatotroph tumour component (Fig. 1). Immunolabelling for the alpha-subunit of glucoprotein hormones was strong in a large proportion of ganglionic cells in cases 2–4 and was also observed in a few ganglionic cells in patient 1. Only a few neuroendocrine tumour cells in the tumour tissue in patients 2–4 and none in patient 1 expressed the alpha-subunit. (Supplemental Fig. 1). PIT1 was positive not only in the somatrotroph tumour cells in patients 2–4 but also in a small proportion of neuronal cells in the gangliocytoma component in all cases, including, interestingly, the specimen from patient 1 with a corticotroph tumour (Fig. 2). A dot-like cytokeratin Cam5.2 pattern depicted the sparsely granulated subtype of somatotroph tumour in all three cases with acromegaly. Surprisingly, fibrous body-like Cam5.2 positive inclusions were also observed in a few neuronal cells in patients 2–3. In patient 4, both fibrous bodies and diffuse intracytoplasmic Cam5.2 staining were observed in a significant proportion of ganglionic cells. There were no Cam 5.2 positive cells in either ganglionic or neuroendocrine tumour component in the specimen from patient 1. (Supplemental Fig. 2). The proliferation index was low, except in patient 2, where the Ki67 index reached 5% in the PitNET component. The Ki67 index in the gangliocytoma component in all tumours was < 1%.

**Fig. 3:**
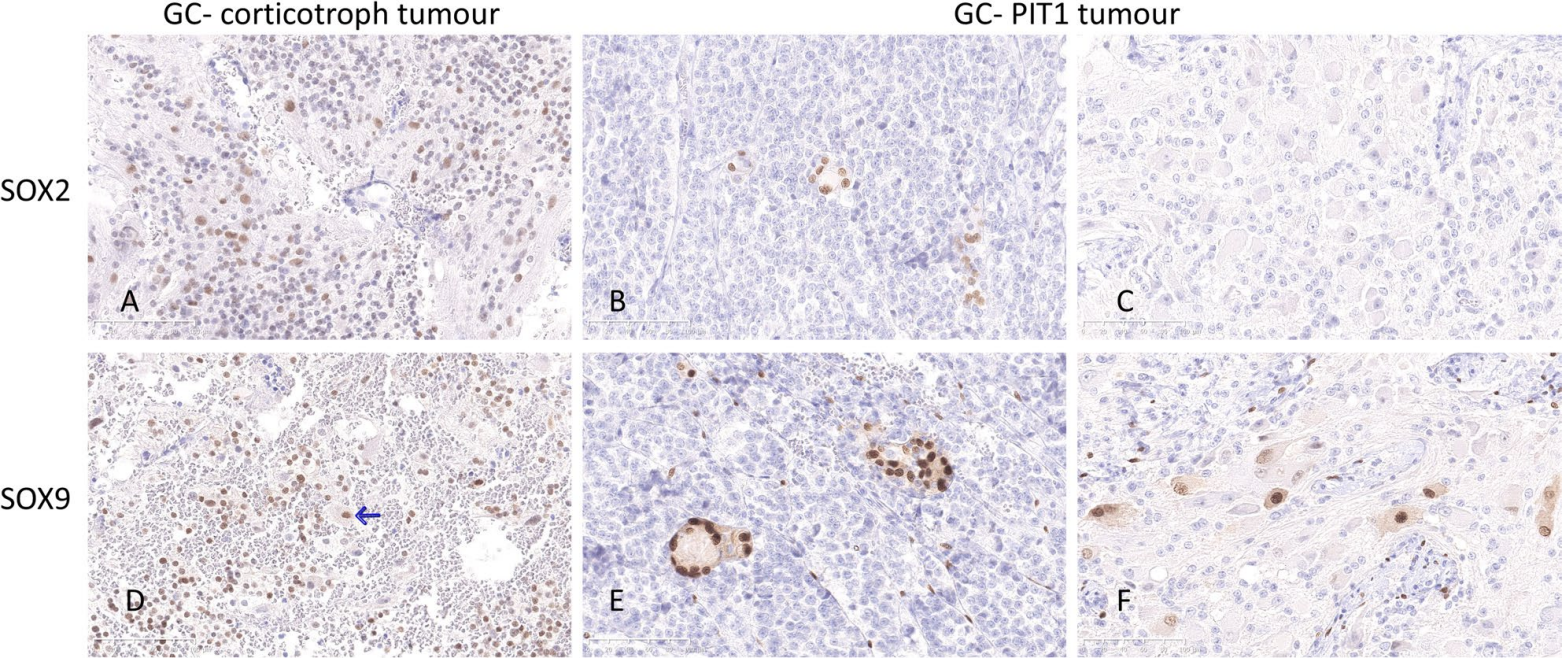
Stem cell marker SOX2 (upper row) was expressed in a subset of cells within both the neuroendocrine and ganglionic components in the mixed GC-corticotroph tumour in patient 1 (**A**). In the GC-PIT1 tumour in patient 3, SOX2 was positive in a few, frequently grouped cells within the neuroendocrine tumour component (**B**), but not in the ganglionic cells (**C**). SOX9 (lower row) was expressed in a subset of cells within the neuroendocrine tumour component in patient 1 (**D**), and only in a few ganglionic cells (arrow). In the tumour from patient 3, SOX9 was distinctly positive in small groups of cells (niches) within the somatotroph tumour (**E**) and in a subset of ganglionic cells (**F**). (Magnification 200x for all microphotographs)

SOX2, SOX9 and PROP1 were expressed in a subset of cells in non-neoplastic adenohypophysial tissue. When present, epithelial cystic remnants of Rathke’s pouch expressed SOX2 and SOX9, but not PROP1. In addition, immunolabelling of SOX9, a regulator of stem cell and supportive (niche) stem cell programs, was observed in a subset of ganglionic cells and in scattered or small groups of cells within the somatotroph tumours of patients 2–4. In the mixed GC-corticotroph tumour in patient 1, SOX9 immunolabelling was observed, albeit with reduced intensity, in a significant proportion of cells within the corticotroph tumour component and in scattered ganglionic cells. SOX2 was also positive in a subset of ganglionic cells and in scattered cells within the corticotroph tumour component in patient 1. In patients 2–4 with mixed GC-somatotroph tumour, SOX2 was positive in a few cells within the somatotroph tumours but not in the ganglionic cells. PROP1, an early embryonic pituitary cell progenitor, was not expressed in any of the tumour specimens. (Fig. 3)

**Fig. 4:**
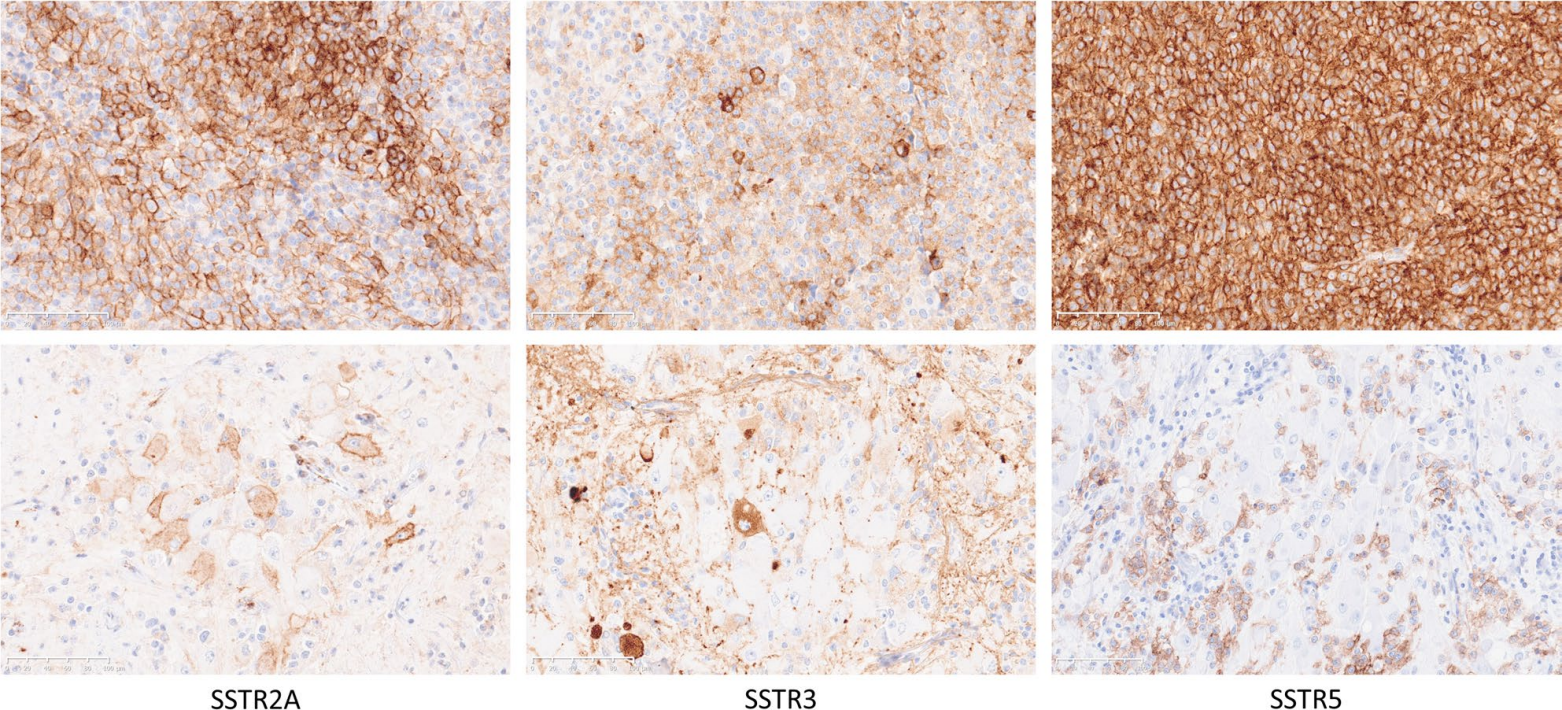
Immunolabelling of SSTR type 2A, 3 and 5 in PitNET (upper row) and gangliocytoma (lower row) component of the tumour in patient 3 with a mixed GC-PIT1 tumour. (Magnification 100x for microphotographs in the upper row and 200x for the microphotographs in the lower row)

There was a relatively high expression of SSTR2 (IRS 6–9) and SSTR5 (IRS 6–12) in the somatotroph tumours, and a high SSTR3 expression in the corticotroph tumour (IRS 12). SSTR1 and 3 were sparsely positive in ganglionic tumour cells in patient 1. In patients with acromegaly, SSTRs demonstrated variable immunolabelling in the gangliocytoma component, with SSTR1 being strongly positive (IRS 12) in patients 2 and 4, and SSTR2, 3 and 5 being variably positive (IRS 2–6). SSTRs expression in PitNET and GC component in patient 3 is illustrated in Fig. 4.

Detailed immunohistochemical results for all tumours, both neuroendocrine and gangliocytoma components, are presented in Table 2.

**Table 2:** Immunohistochemical analyses of PitNET and gangliocytoma component from four patients with mixed gangliocytoma—PitNET; one patient with Cushing’s disease (Patient 1) and three patients with acromegaly (Patients 2–4)

	Patient 1	Patient 2	Patient 3	Patient 4
*PitNET component*				
TPIT	+	−	−	−
SF1	−	−	−	−
PIT1	−	+	+	+
CAM5.2	−	+ (fibrous bodies)	+ (fibrous bodies)	+ (fibrous bodies)
Ki67	1%	5%	1%	1%
ACTH	+	−	−	−
GH	−	+	+	+
PRL	−	+	+	+ (a few cells)
TSH	−	−	−	−
FSH/LH	−	−	−	−
Alpha-HCG	−	+/−	+/−	+/−
SSTR1	0 × 0 = 0	0 × 0 = 0	3 × 3 = 9	1 × 1 = 1
SSTR2A	0 × 0 = 0	3 × 3 = 9	3 × 2 = 6	3 × 3 = 9
SSTR3	4 × 3 = 12	2 × 2 = 4	3 × 2 = 6	4 × 2 = 8
SSTR5	0 × 0 = 0	3 × 2 = 6	4 × 3 = 12	4 × 3 = 12
SOX2	+ in a few cells	+ in a few cells	+ in a few cells	+ in a few cells
SOX9	+ in a subset of cells	+ in a few cells	+ in a few cells	+ in a few cells
PROP1	−	−	−	−
*Gangliocytoma component*				
TPIT	−	−	−	−
SF1	−	−	−	−
PIT1	+/−	+/−	+/−	+/−
CAM5.2	−	+/−	+/−	+/−
Ki67	1%	1%	1%	1%
ACTH	−	−	−	−
GH	−	+/−	+/−	+/−
PRL	−	+/−	+/−	+/−
TSH	−	−	−	−
FSH/LH	−	−	−	−
Alpha-HCG	+/−	+	+	+
SSTR1	1 × 1 = 1	4 × 3 = 12	2 × 2 = 4	4 × 3 = 12
SSTR2A	0 × 0 = 0	2 × 1 = 2	2 × 3 = 6	3 × 2 = 6
SSTR3	1 × 3 = 3	2 × 3 = 6	3 × 2 = 6	2 × 3 = 6
SSTR5	0 × 0 = 0	1 × 1 = 1	1 × 2 = 2	2 × 2 = 4
SOX2	+ in a subset of cells	−	−	−
SOX9	+ in a few cells	+ in a subset of cells	+ in a subset of cells	+ in a subset of cells
PROP1	−	−	−	−

### Genome-wide expression profiling

**Fig. 5:**
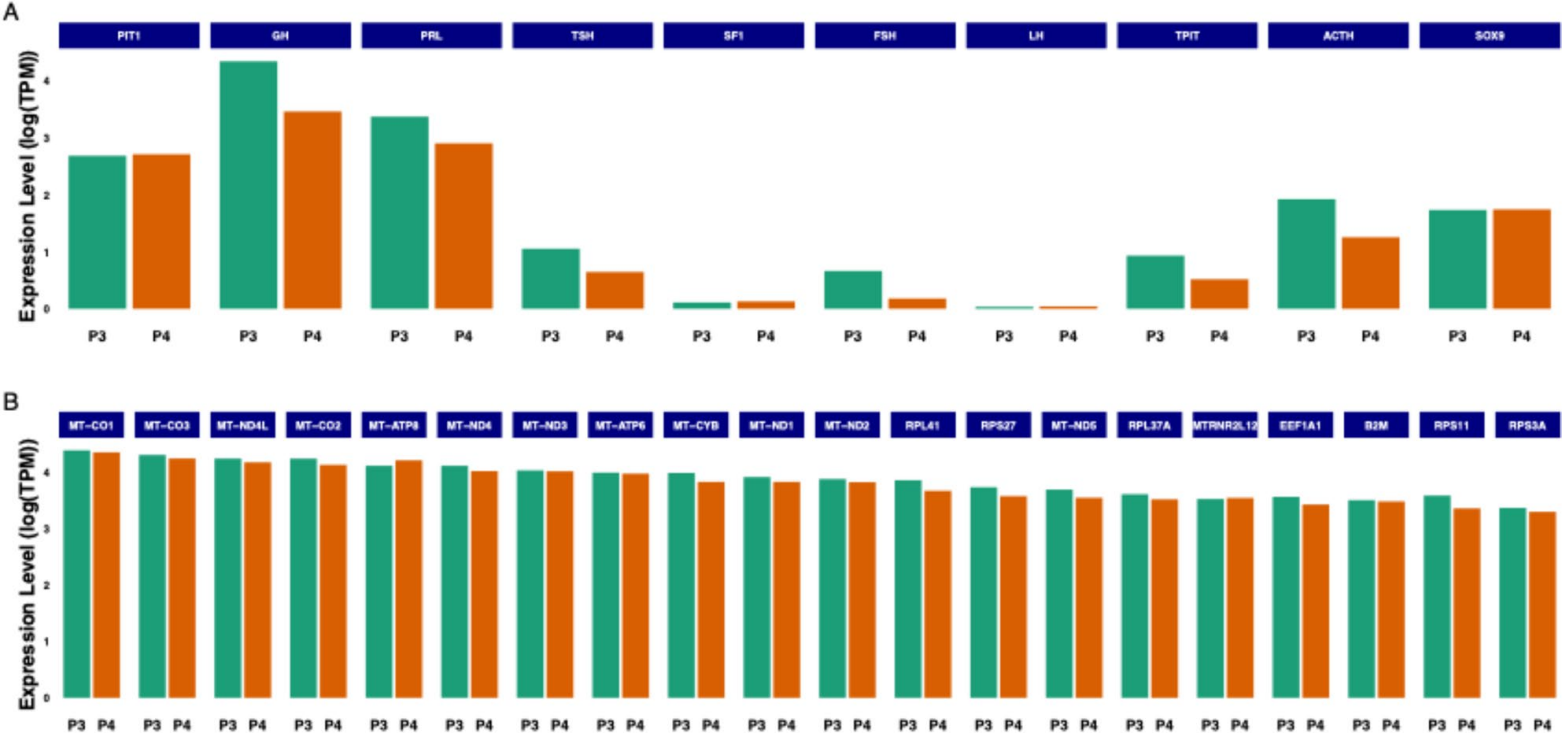
Transcriptomic results showing the expression levels for the pituitary-specific genes and SOX9 (Fig. 4A) and the top 20 mostly expressed genes (Fig. 4B) in two GC-somatotroph tumours (Patient 3 and 4)

Whole genome mRNA sequencing, performed on two specimens representing mixed gangliocytoma-somatotroph tumour, revealed, as expected, high mRNA levels for PIT1, GH and PRL, in concordance with the immunohistochemical results. A moderate expression level of ACTH and TPIT was observed, whereas the gonadotroph markers, SF1, FSH and LH were sparsely expressed. Interestingly, *SOX9 (SRA1)* was expressed at a relatively high level, but not the other two stemness-related genes (*SOX2* and *PROP1*). mRNA expression levels for specific genes were similar in the two tumours. Thirteen of the 20 upregulated genes coded for mitochondrial proteins: MT-CO1-3 belonging to the cytochrome C oxidase complex, MT-ATP 6 and 8 regulating ATP synthesis, MT-ND1-4, 4 L, 5 representing core units of the respiratory chain responsible for transferring electrons from NADH to the respiratory chain. The mitochondrial proteins MT-CYB and MT-RNR2L12 have neuroprotective and antiapoptotic roles. Another group of upregulated genes belong to the ribosomal proteins (RPL41 and 37A; RPS3A, 11, and 27), and EEF1A1, involved in the enzymatic delivery of aminoacyl tRNA to the ribosomes during protein synthesis. Information on the respective genes were summarised from the Human Protein Atlas (www.proteinatlas.org). Transcriptomics results, showing expression levels of pituitary gland-specific genes, SOX9 and the top 20 mostly expressed genes in two GC-somatotroph tumours, are presented in Fig. 5 and Supplemental Table 2. GSEA revealed enrichment of oxidative phosphorylation and translation-related pathways among top 20 genes. Results are presented in Supplemental Table 3.

### Target enrichment next-generation sequencing

No pathogenic or likely pathogenic variants were detected in any of the tumours. TMB was low (score 0). There was no evidence of MSI, except for the tumour tissue from patient 1 where MSI index was 7.4%, which is slightly above the cut off of 6%.

### Genome-wide DNA methylation profiling

Three of the four tumours (patients 2–4) matched with score 0.99 to the entity “PitNET, somatotropin (STH)-Subtype, Subclass Sparsely Granulated” by CNS tumour classifier v12.8 using 850k methylation profiling for classification. No scores towards the two other STH-producing entities, “PitNET, STH-Subtype, Subclass Densely Granulated A/B”, were observed. This emphasises a strictly converging and clear classifier prediction result by v12.8 for these three cases. In contrast, the CNS tumour classifier v12.8 with a score of 0.89 strongly favoured and almost classified the tumour from patient 1 as methylation class “PitNET, ACTH Subtype”. Chromosomal alterations were not identified from the CNV profiles in any of the four tumours. (Supplemental Fig. 3).

## Discussion

We present clinical, immunohistochemical, targeting DNA gene sequencing and methylome data on four patients with hormone-producing mixed GC-pituitary neuroendocrine tumours, three patients with acromegaly and one with Cushing’s disease. For two patients with acromegaly transcriptomic data were also available. Despite being based on a small series of these uncommon tumours, our findings indicate differences in clinical course, protein expression patterns and molecular features between mixed GC-PitNETs and respective PitNETs not associated with a gangliocytoma. The expression pattern of selected stem cell markers further supports the previously proposed common origin of neuroendocrine and ganglionic tumour components and involvement of stem cells in tumorigenesis.

All patients with acromegaly in our series had sparsely-granulated somatotroph tumour, consistent with previous data [2, 3, 5, 28]. We had the opportunity to study a patient with Cushing’s disease caused by a corticotroph tumour as part of a mixed GC-PitNET. Mixed GC–PitNETs causing Cushing’s disease are uncommon, with only 18 cases previously reported [2, 6–9]. Our patient developed clinical features of Cushing’s disease many years after the initial diagnosis, which possibly depends on the progress of the tumour remnant over the years.

Although based on only three cases, the acromegaly patients in our cohort seem to be more therapy-resistant than patients with isolated somatotroph tumours [29]. None of our patients responded effectively to SSAs, despite high expression of either SSTR2 or SSTR5 or both in the PitNET component, and one patient showed tumour progression under SSA treatment. All patients required treatment with GH receptor antagonist (Pegvisomant), one patient required secondary surgery, and two patients underwent radiotherapy to achieve disease control. All three patients had a sparsely-granulated somatotroph tumour component, which could be a possible explanation for the unsatisfactory effect of SSAs. Sparsely-granulated tumours account for 15–35% of patients with acromegaly [30] and seem to be more resistant to treatment with SSAs compared to densely-granulated tumours [30, 31], presumably because of lower expression of SSTR2 [30, 32], although other mechanisms for resistance to SSA cannot be excluded. In addition to the sparsely-granulated pattern, it has previously been speculated that paracrine secretion of hormone-stimulating factors from the residual gangliocytic component may be one of the reasons for a worse response to SSAs in patients with mixed GC-PitNET [2].

 While the correlation between SSTRs and the response to SSA treatment is well explored in patients with somatotroph tumours [11–13, 32], it is not known whether the presence and distribution of SSTRs in the ganglionic component of mixed GC-PitNET may have an impact on the therapeutic response. Positive staining for SSTRs has been demonstrated in a patient with thyrotropin/thyrotropin-releasing hormone (TSH/TRH)-producing isolated pituitary gangliocytoma [33]. However, there is no data on SSTR expression in the ganglionic cells of mixed GC-PitNETs. Our three acromegaly patients had a relatively high SSTR2 and SSTR5 expression in somatotroph tumour cells compared to a lower expression in the ganglionic cells, which suggests that SSTR expression in the ganglionic component may, at least to a certain degree, also determine the tumour’s response to treatment with SSAs.

Previously, it was demonstrated that SSTR2 expression was inversely correlated with the dose of the GH receptor antagonist Pegvisomant required to achieve biochemical remission [34]. This is supported by the results in our cohort, where patient 4, having a moderate to high SSTR2A expression in both the ganglionic and neuroendocrine tumour cells, required a significantly lower Pegvisomant dose than patient 3, who had a lower expression of SSTR2A in somatotroph tumour cells. Although based on only three cases, it seems that Pegvisomant should be considered early when the SSA effect is unsatisfactory or lacking in patients with mixed GC-somatotroph tumours.

To explore the potential role of pituitary transcription factors and stem cells in the histopathogenesis of mixed GC-PitNETs, we performed immunohistochemical analysis with PIT1, TPIT, stem cell markers SOX2, and SOX9, as well as a progenitor marker PROP1. A minor proportion of ganglionic cells in mixed GC-somatotroph tumours in our cohort, similar to previous reports [18, 35], expressed PIT1. Surprisingly, PIT1 was even positive in a few ganglionic cells in the mixed GC-corticotroph tumour in patient 1. Thus, our data support the previous hypothesis that trans-differentiation of neuroendocrine cells plays a role in the development of the neuronal tumour component. PIT1 seems to be particularly important in this process, even beyond the pituitary cell lineage differentiation of the neuroendocrine tumour component, as we demonstrated PIT1 expression in ganglionic cells also in the gangliocytoma associated with a corticotroph tumour. Additional markers frequently associated with, though not specific for PIT1 cell lineage, were preserved in ganglionic cells. Alpha-subunit of glycoprotein hormones was present in all four tumour specimens in our study, and cytokeratin Cam5.2 was present in ganglionic cells in patients with acromegaly, which is in accordance with a recent report [9].

Stem cell markers SOX2 and SOX9, and a progenitor cell marker PROP1, were expressed, as expected, in a subset of cells in non-neoplastic adenohypophysial tissue. The former two were also positive in epithelial rests of Rathke’s pouch embedded in the tumour tissue. In addition, SOX2 immunolabelling was observed in scattered or small groups of cells within the neuroendocrine tumour component in all four cases, similarly to the pattern previously reported in gonadotroph tumours [21]. SOX2 was also expressed in a few cells with ganglionic characteristics in the mixed GC-corticotroph tumour in our cohort. Interestingly, SOX9 was distinctly positive in a subset of ganglionic cells and within the population of the neuroendocrine cells in all four cases. In patients with acromegaly, SOX9 positive cells in the somatotroph component were frequently aggregated in small groups, which could potentially correspond to the stem cell niches reported previously [36]. SOX9 was also among the genes upregulated in two mixed GC-somatotroph tumours we had opportunity to study using global mRNA analysis. The expression of stem cell markers, in particular SOX9, which was distinctly positive in both tumour components, indicates a common origin of neuroendocrine and ganglionic cells in mixed GC-PitNETs and further supports the role of stem cells in the development of this uncommon tumour type. SOX9 expression in combination with a lack of PROP1 argues for activation of the stem cell/supportive stem cell programme and against reversion to early embryonic progenitors.

Transcriptomic data on two cases of mixed GC-somatotroph tumours in our study revealed the activation of genes involved in mitochondrial and ribosomal mechanisms. Interestingly, except for PIT1 lineage-related genes, none of the top 20 genes with high expression levels overlapped with the previously described top genes related to the PIT1 cell lineage [22]. Although based on only two cases, these data may indicate a distinct gene expression pattern in somatotroph tumours occurring as a part of mixed GC-PitNET. Disturbances in the mitochondrial genes involved in the cytochrome C oxidase complex, as well as ATP metabolism and electron transfer to the respiratory chain, play a role in different malignancies, including brain [37], gastric [38] and breast tumours [39]. However, there are no reports on the dysregulation of these mitochondrial genes in the pituitary tumours. Several of the upregulated genes in our two mixed GC-PitNETs are known as tumorigenic and prognostic factors in malignant tumours, e.g. ribosomal protein RPS3A in hepatocellular carcinoma [40] and EEF1A1 in colorectal carcinoma [41]. Moreover, the upregulated ribosomal genes may influence therapeutic response. For example, RPL41 increases the sensitivity of retinoblastoma cells to chemotherapeutic drugs [42], and EEF1A1 is a promising target for drugs affecting the EEF1A1/MAPK axis [41]. Further studies on these novel therapeutic markers in pituitary tumours and in a larger number of mixed GC-PitNETs are warranted.

Similarly to majority of benign PitNETs, TMB was low and there were no pathogenic or likely pathogenic variants identified in target enrichment DNA sequencing panel covering more than 500 cancer-related genes.

The methylomes of the four cases were directed towards “Tumours of the sellar region” and further to “Pituitary adenoma” by the classifier algorithm hierarchy, as expected. Tumours from patients 2–4 were classified as “Pituitary adenoma, subtype GH-producing, subclass sparsely granulated” based on 850k methylation profiling. The tumour from patient 1, on the other hand, deviated in this regard with a strong classification trend towards the methylation class “Pituitary adenoma, subtype ACTH-producing”. With a detection rate of 90% and an incomplete 850k dataset for this sample, technical parameters could influence the classification outcome. Among the three provisional subclass members of the methylation family Pituitary adenoma, GH producing, GC-somatotroph tumours in our cohort showed no or minimal score values towards “Pituitary adenoma, subtype STH-producing, subclass densely granulated A or B”. This emphasises a strong determination in the classifier prediction for the sparsely-granulated subclass over the densely-granulated subclasses A/B by the recent v12.8 of the Brain tumour classifier algorithm for these three cases.

Gangliocytoma does not exist as a separate methylation class entity in the classifier tool and is not recognised by the algorithm [43]. Although the potential contribution from neoplastic ganglion cells in the methylomes of the four mixed gangliocytoma-PitNET tumours is not easily accessible for evaluation, it did not confuse the classifier tool. The DNA methylation-based classification output was in concordance with the hormone-secreting profiles. A fingerprint of the neoplastic ganglion cells in the methylomes of mixed GC-PitNET tumour warrants further in-depth comparative bioinformatic studies of the DNA methylation profiles from gangliocytomas, mixed GC-PitNETs and PitNETs.

The four mixed GC-PitNETs examined were very similar with respect to CNV profiles, as no chromosomal imbalances were detected. Thus, the four tumours could not be distinguished based on their individual CNV profiles. In comparison, chromosomal imbalances can be observed in cases matching the methylation classes “Pituitary adenoma, ACTH-producing” or “Pituitary adenoma, STH producing”, with a higher frequency of CNV alterations affecting whole chromosomes in the latter, as outlined in the class descriptions of the v12.8 classifier tool. The CNV profiles of gangliocytomas are expected to show a flat line. Therefore, a CNV trait from the neoplastic ganglion cells would not be discernible in the CNV profiles from the mixed GC-PitNETs.

A limitation of the study is that it includes only four GC-PitNET samples. However, the rarity of this tumour type is a limiting factor that hampers studies involving larger tumour cohorts. Of note, as transcriptomic analyses rely on bulk RNA sequencing from heterogeneous tumour samples, the variation in cellular composition may partially confound the observed gene expression differences. Whole single-cell/single-nucleus genetic and epigenetic profiling would optimally disentangle the ganglionic and neuroendocrine compartments of GC-PitNETs. However, the rarity of the tumour type and tissue constraints make single-cell/single-nucleus analyses difficult.

## Conclusions

Based on a small series of four patients, it appears that patients with acromegaly caused by somatotroph tumour as a part of mixed GC-PitNETs are more resistant to pharmacological therapy compared to patients with somatotroph tumours not associated with gangliocytoma. This resistance may partly be related to the sparsely-granulated phenotype and partly to the still unclear impact of ganglionic cells in mixed GC-PitNETs. The expression of PIT1 and stem cell markers, especially SOX9, in both ganglionic and neuroendocrine tumour cells further supports the common origin of the two tumour components of mixed GC-PitNETs. PIT1 seems to be involved in the genesis of GC-PitNET beyond cell lineage differentiation, as immunolabelling of PIT1 and other PIT1 cell lineage markers was present in ganglionic cells even in the gangliocytoma associated with the corticotroph tumour. Unique transcriptomic data, although based on only two cases, suggest a distinct gene expression pattern compared to pure somatotroph tumours, with the involvement of mitochondrial and ribosomal genes. Some of these genes may be potential prognostic and novel therapeutic markers. The methylation profile of mixed GC-PitNET is similar to PitNETs, allowing for classification based on the secretory type.

## Supplementary Information

Below is the link to the electronic supplementary material.

Supplemental Table 1. Summary of the antibodies used for the immunohistochemical analyses

Supplemental Table 2. Expression levels (TPM) of all genes in the two cases of mixed GC-somatotroph tumours

Supplemental Table 3. Gene set enrichement analysis results

Supplemental Table 4. A list of 544 genes covered by the GMS560 gene panel used in the targeting DNA sequencing analysis

Supplemental Figure 1. Immunolabelling for the alpha-subunit of glucoprotein hormones present in the majority of ganglionic cells and in scattered neuroendocrine tumour cells in the tumour specimen from a patient with mixed gangliocytoma-somatotroph tumour (A). Only a few ganglionic cells were positive in the mixed GC-corticotroph tumour from Patient 1. (Magnification 200×)

Supplemental Figure 2. A dot-like cytokeratin Cam5.2 pattern, characteristic of the sparsely granulated subtype of somatotroph tumour, was observed in all three cases with acromegaly (A) and was even observed in a few neuronal cells in patients 2–3 (B). Both fibrous bodies and diffuse intracytoplasmic Cam5.2 staining were observed in the ganglionic cells in patient 4 (C). Cam5.2 was negative in both ganglionic and neuroendocrine tumour components in the mixed GC-corticotroph tumour (D). (Magnification 200×)

Supplemental Figure 3. CNV data derived from genome-wide methylation analysis revealed flat profiles in patients 1-4 (A-D)

## Data Availability

Original data generated and analysed during this study are included in this published article and the supplemental material.

## Abbreviations

ACTH: Adrenocorticotropic hormone
BAM: Binary Alignment Map
CNV: Copy number variation
FFPE: Formalin-fixed paraffin-embedded
FSH: Follicle-stimulating hormone
GC: Gangliocytoma
GC-PitNET: Gangliocytoma-pituitary neuroendocrine tumour
GH: Growth hormone
GMS: Genomic Medicine Sweden
GSEA: Gene Set Enrichment Analysis
HCG: Human chorionic gonadotropin
HE: Haematoxylin-eosin
IGF-1: Insulin-like Growth Factor 1
IGV: Integrated Genomics Viewer
IHC: Immunohistochemical
INDELS: Insertions and deletions
IRS: Immunoreactive score
LH: Luteinizing hormone
Mb: Megabase
MSI: Microsatellite instability
PANCH: Pituitary adenoma-neuronal choristoma
PIT1: Pituitary specific positive transcription factor 1
PitNET: Pituitary neuroendocrine tumour
PRL: Prolactin
PROP1: PROP paired-like homeobox 1
QCI: Clinical Insight Interpret
SF1: Steroidogenic factor 1
SNVs: Single nucleotide variants
SOX: Sex-determining region Y-box
SSA: Somatostatin analogue
SSTR: Somatostatin receptor
STH: Somatotropin
TMB: Tumour mutational burden
TPIT: T-box transcription factor
TPM: Transcript per million
TRH: Thyrotropin-releasing hormone
TSH: Thyrotropin
VAF: Variant allele frequency

## Notes

Olivera Casar-Borota and Oskar Ragnarson are joint senior authors.
