# Supplementary material for: Mixed gangliocytoma-pituitary neuroendocrine tumour: clinical, immunohistochemical, and molecular genetic profiles in a series of four patients
Source: Acta Neuropathol Commun. 2026 Jan 30;14:48. doi: 10.1186/s40478-026-02225-x (PMC12930862; doi:10.1186/s40478-026-02225-x)
Supplement: Supplementary file 1 — Supplemental Table 1. Summary of the antibodies used for the immunohistochemical analyses [file 40478_2026_2225_MOESM1_ESM.docx]

**Supplementary table 1**. Summary of the antibodies used for the immunohistochemical analyses.

| **Antibody** | **Antibody clonality** | **Clone** | **Supplier (cat.nr)** | **Dilution** | **Platform** |
| --- | --- | --- | --- | --- | --- |
| anti-FSH | MAB | C10 | DAKO (M3504), RRID: AB_2079146 | 1:300 | DAKO Autostainer  En Vision Flex |
| anti-LH | MAB | 93C | DAKO (M3502), RRID: AB_2135325 | 1:400 | DAKO Autostainer  En Vision Flex |
| anti-TSH | MAB | 0042 | DAKO (M3503),  RRID: AB_2287785 | 1:100 | DAKO Autostainer  En Vision Flex |
| anti-GH | PAB | - | DAKO (A0570),  RRID: AB_2617170 | 1:3000 | DAKO Autostainer En Vision Flex |
| anti-ACTH | MAB | 02A3 | DAKO (M3501),  RRID: AB_2166039 | 1:1200 | DAKO Autostainer En Vision Flex |
| anti-PIT1 | PAB | - | Novus Biologicals (NBP1-92273),  RRID: AB_11030310 | 1:500 | DAKO Autostainer En Vision Flex |
| anti-SF1 (NR5A1) | MAB | N1665 | ThermoFisher/TF Scientific (434200),  RRID: AB_2532209 | 1:100 | DAKO Autostainer En Vision Flex |
| anti-TPIT (TBX19) | MAB | CL6251 | Atlas Antibodies (AMAb91409),  RRID: AB_2716678 | 1:1000 | DAKO Autostainer En Vision Flex |
| anti-Ki67 | MAB | MIB1 | DAKO (IR626/GA626), RRID: AB_2890068/ AB_2687921 | ready-to-use | DAKO Autostainer En Vision Flex |
| anti-SSTR1 | MAB | UMB-7 | Abcam (ab137083), RRID: N/A | 1:100 | DAKO Autostainer En Vision Flex |
| anti-SSTR2A | MAB | UMB-1 | Abcam (ab134152), RRID: AB_2737601 | 1:1000 | DAKO Autostainer En Vision Flex |
| anti-SSTR3 | MAB | UMB-5 | Abcam (ab137026), RRID: N/A | 1:4000 | DAKO Autostainer En Vision Flex |
| anti-SSTR5 | MAB | UMB-4 | Abcam (ab109495), RRID: AB_10859946 | 1:750 | DAKO Autostainer En Vision Flex |
| anti-SOX2 | MAB | SP76 | Cell Marque (371R-15), RRID: N/A | 1:50 | TF Lab Vision Autostainer 480S Module |
| anti-SOX9 | MAB | CL0639 | Atlas Antibodies (AMAb90795),  RRID: AB_2665670 | 1:500 | ThermoFisher Lab Vision Autostainer 480S Module |
| anti-PROP1 | PAB | - | Atlas Antibodies (HPA049839),  RRID: AB_2732557 | 1:150 | ThermoFisher Lab Vision Autostainer 480S Module |

cat.nr: catalogue number

MAB: Monoclonal antibody

N/A: not available

PAB: Polyclonal antibody

RRID: Research Resource Identifier.
