# Supplementary figures and images for: Mixed gangliocytoma-pituitary neuroendocrine tumour: clinical, immunohistochemical, and molecular genetic profiles in a series of four patients
Source: Acta Neuropathol Commun. 2026 Jan 30;14:48. doi: 10.1186/s40478-026-02225-x (PMC12930862; doi:10.1186/s40478-026-02225-x)

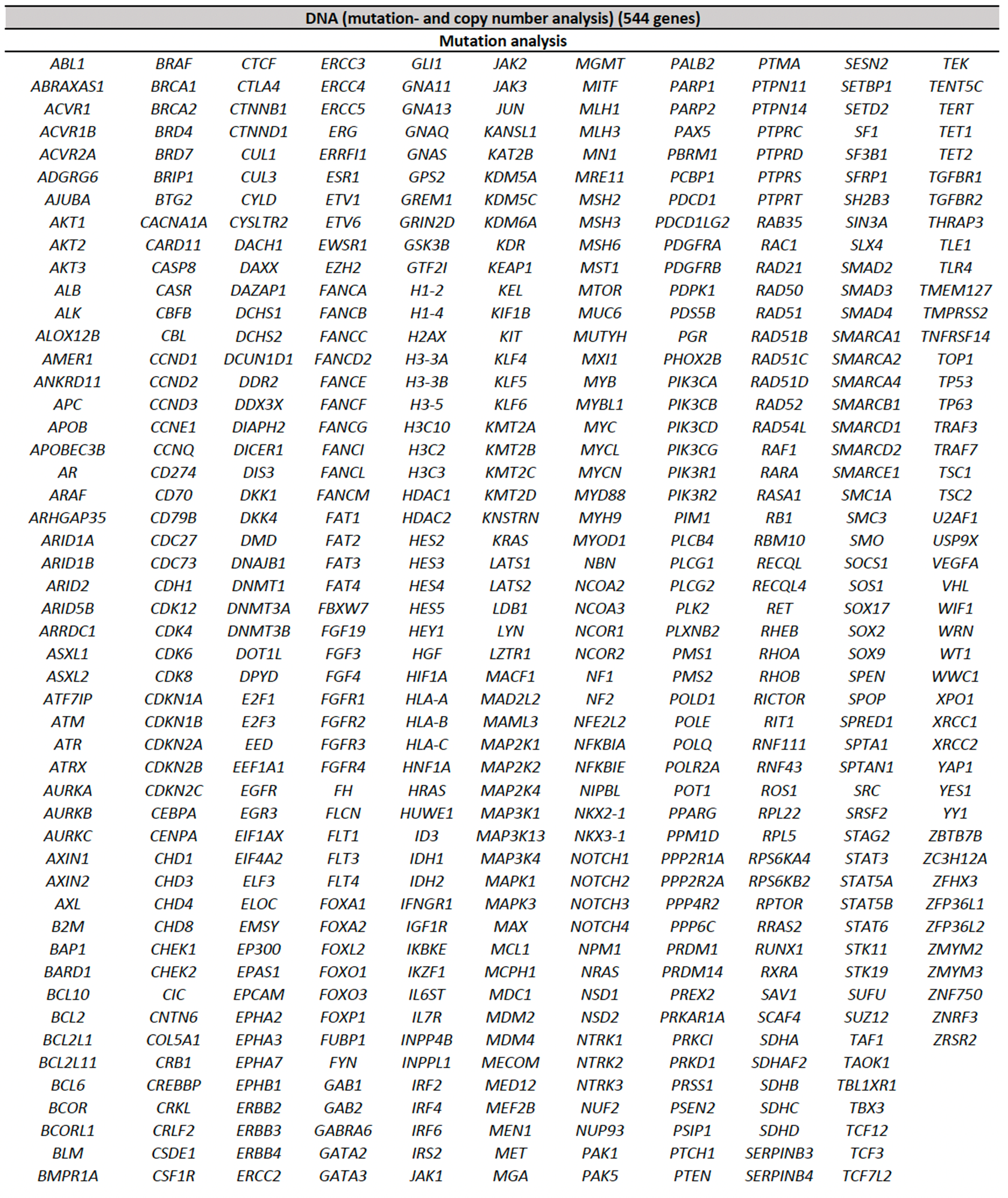

Supplement: Supplementary file 4 — Supplemental Table 4. A list of 544 genes covered by the GMS560 gene panel used in the targeting DNA sequencing analysis [file 40478_2026_2225_MOESM4_ESM.png]

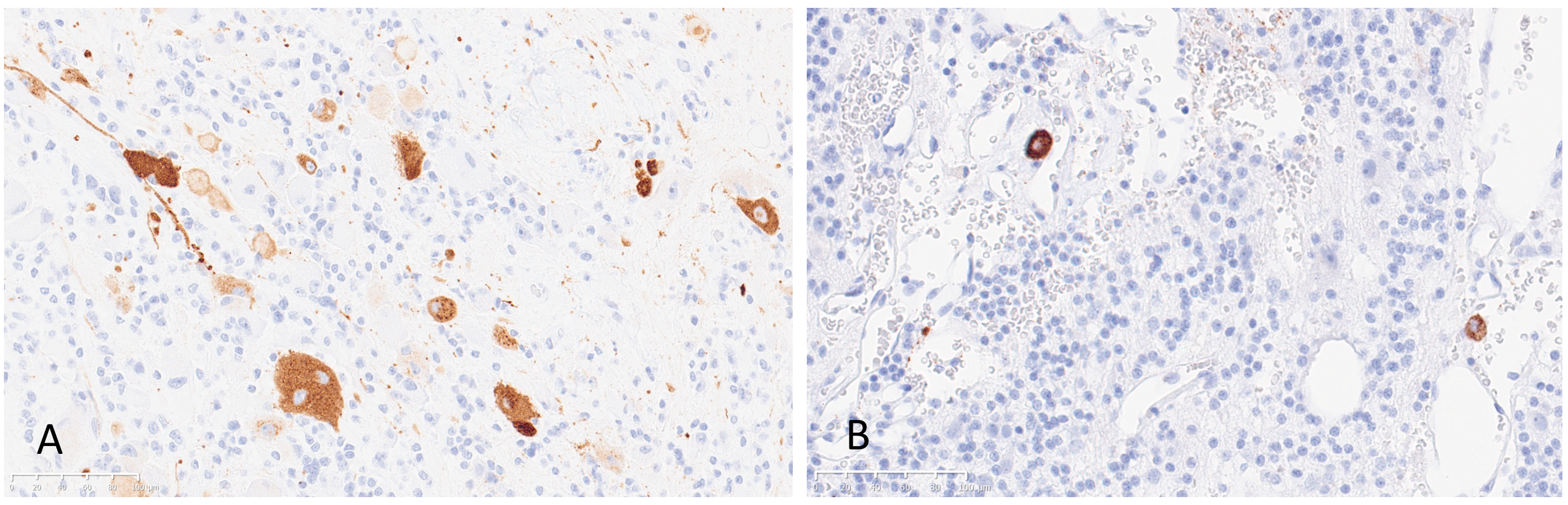

Supplement: Supplementary file 5 — Supplemental Figure 1. Immunolabelling for the alpha-subunit of glucoprotein hormones present in the majority of ganglionic cells and in scattered neuroendocrine tumour cells in the tumour specimen from a patient with mixed gangliocytoma-somatotroph tumour (A). Only a few ganglionic cells were positive in the mixed GC-corticotroph tumour from Patient 1. (Magnification 200×) [file 40478_2026_2225_MOESM5_ESM.png]

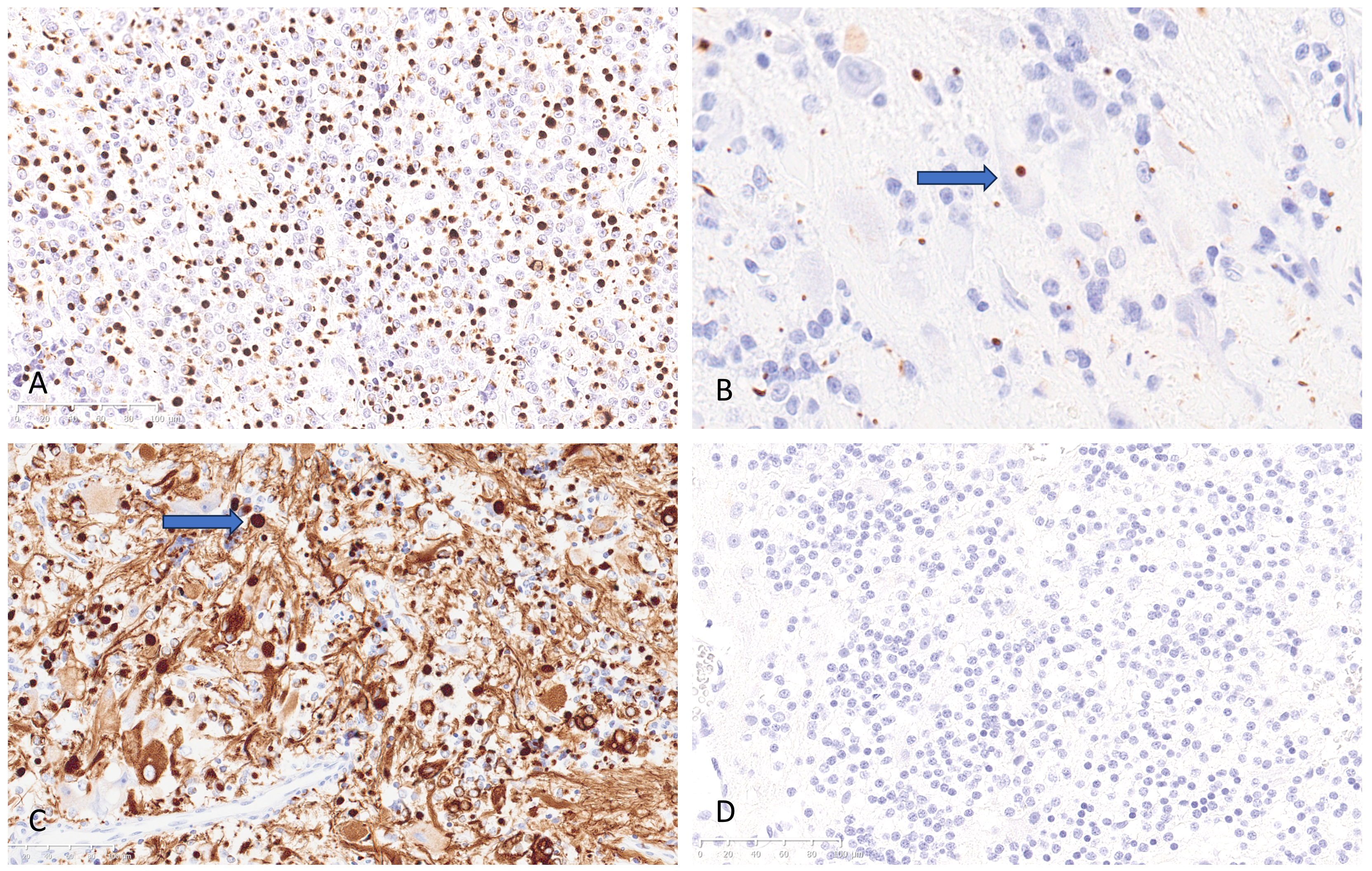

Supplement: Supplementary file 6 — Supplemental Figure 2. A dot-like cytokeratin Cam5.2 pattern, characteristic of the sparsely granulated subtype of somatotroph tumour, was observed in all three cases with acromegaly (A) and was even observed in a few neuronal cells in patients 2–3 (B). Both fibrous bodies and diffuse intracytoplasmic Cam5.2 staining were observed in the ganglionic cells in patient 4 (C). Cam5.2 was negative in both ganglionic and neuroendocrine tumour components in the mixed GC-corticotroph tumour (D). (Magnification 200×) [file 40478_2026_2225_MOESM6_ESM.png]

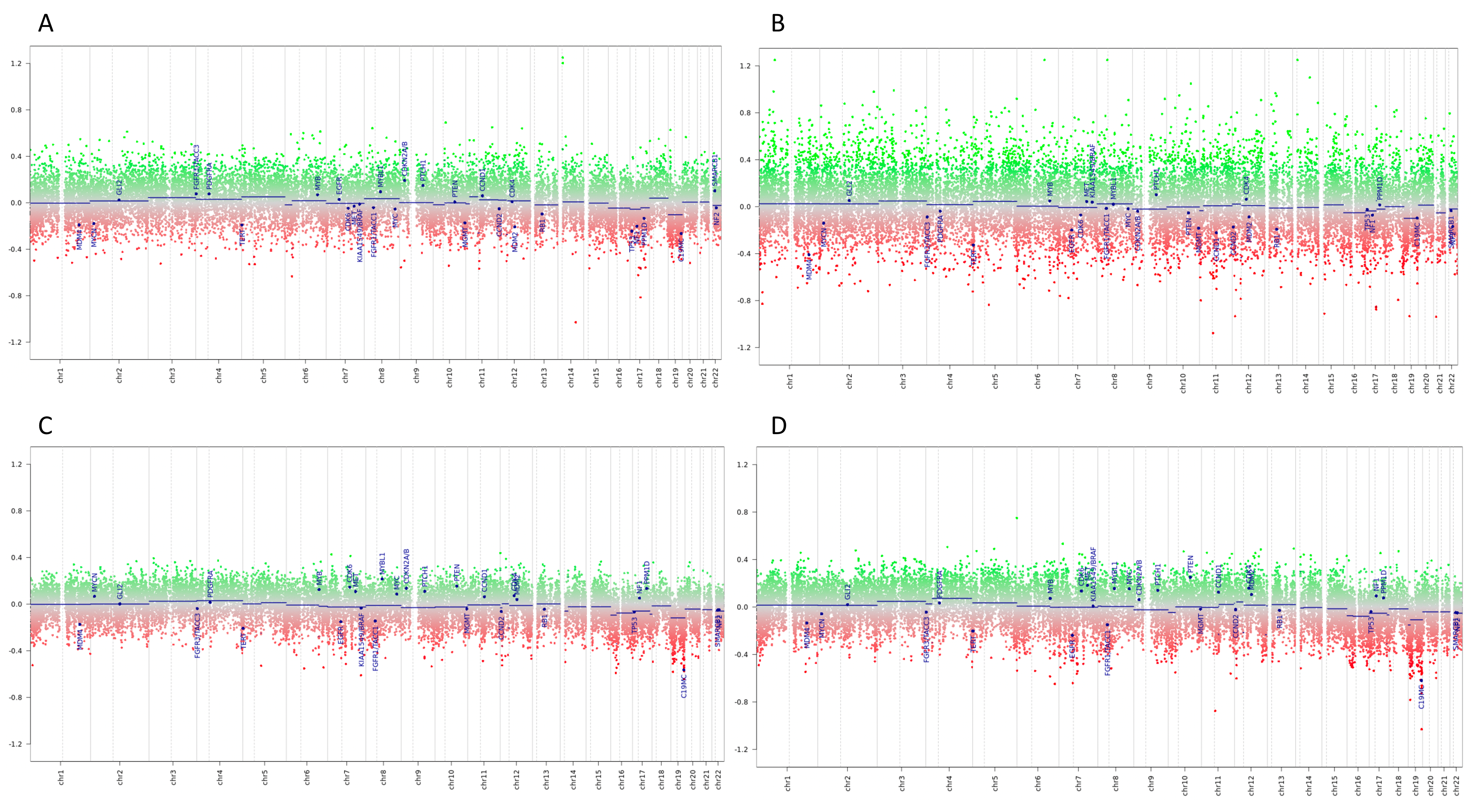

Supplement: Supplementary file 7 — Supplemental Figure 3. CNV data derived from genome-wide methylation analysis revealed flat profiles in patients 1-4 (A-D) [file 40478_2026_2225_MOESM7_ESM.png]
